# SGLT2 inhibitor treatment is not associated with an increased risk of osteoporotic fractures when compared to GLP-1 receptor agonists: A nationwide cohort study

**DOI:** 10.3389/fendo.2022.861422

**Published:** 2022-08-19

**Authors:** Zheer Kejlberg Al-Mashhadi, Rikke Viggers, Jakob Starup-Linde, Peter Vestergaard, Søren Gregersen

**Affiliations:** ^1^ Steno Diabetes Center Aarhus, Aarhus University Hospital, Aarhus, Denmark; ^2^ Department of Clinical Medicine, Aarhus University, Aarhus, Denmark; ^3^ Steno Diabetes Center North Jutland, Department of Endocrinology, Aalborg University Hospital, Aalborg, Denmark; ^4^ Department of Clinical Medicine, Aalborg University, Aalborg, Denmark; ^5^ Department of Endocrinology and Internal Medicine, Aarhus University Hospital, Aarhus, Denmark

**Keywords:** SGLT2, GLP-1, fracture, diabetes, bone, osteoporosis

## Abstract

**Background:**

Type 2 diabetes mellitus (T2D) is associated with an increased fracture risk. It is debated whether sodium-glucose cotransporter 2 (SGLT2) inhibitors influence fracture risk in T2D. We aimed to investigate the risk of major osteoporotic fractures (MOF) with SGLT2 inhibitors compared to glucagon-like peptide 1 (GLP-1) receptor agonists when used as add-on therapies to metformin.

**Methods:**

We conducted a population-based cohort study using Danish national health registries. Diagnoses were obtained from discharge diagnosis codes (ICD-10 and ICD-8-system) from the Danish National Patient Registry, and all redeemed drug prescriptions were obtained from the Danish National Prescription Registry (ATC classification system). Subjects treated with metformin in combination with either SGLT2 inhibitors or GLP-1 receptor agonists were identified and enrolled from 2012 to 2018. Subjects were then propensity-score matched 1:1 based on age, sex, and index date. Major osteoporotic fractures (MOF) were defined as hip, vertebral, humerus, or forearm fractures. A Cox proportional hazards model was utilized to estimate hazard rate ratios (HR) for MOF, and survival curves were plotted using the Kaplan-Meier estimator.

**Results:**

In total, 27,543 individuals treated with either combination were identified and included. After matching, 18,390 individuals were included in the main analysis (9,190 in each group). Median follow-up times were 355 [interquartile range (IQR) 126-780] and 372 [IQR 136-766] days in the SGLT2 inhibitor and GLP-1 receptor agonist group, respectively. We found a crude HR of 0.77 [95% CI 0.56-1.04] for MOF with SGLT2 inhibitors compared to GLP-1 receptor agonists. In the fully adjusted model, we obtained an unaltered HR of 0.77 [95% CI 0.56-1.05]. Results were similar across subgroup- and sensitivity analyses.

**Conclusion:**

These results suggest that SGLT2 inhibitors have no effect on fracture risk when compared to GLP-1 receptor agonists. This is in line with results from previous studies.

## Introduction

Type 2 diabetes mellitus (T2D) is associated with an increased fracture risk (1) despite normal or even elevated bone mineral density (BMD) levels and higher body mass index (BMI), both of which are protective factors against fracture (2–4).

In the last decades, multiple new glucose-lowering drugs have become available for the management of T2D (5). Sodium-glucose cotransporter 2 (SGLT2) inhibitors and glucagon-like peptide-1 (GLP-1) receptor agonists have recently been recommended for treatment of T2D in subjects with cardiovascular disease (6). In addition, SGLT2 inhibitors are recommended to prevent progression of chronic kidney disease (6). Consequently, the use of these agents is increasing and so is the need for information about potential side effects or impacts on other organs.

Knowledge about the impact of SGLT2 inhibitors and GLP-1 receptor agonists on bone health and fracture risk is limited. Studies have attempted to investigate the effects of various glucose-lowering drugs on fracture risk, although these are generally observational in nature and subject to confounding and insufficient follow-up durations (7). For SGLT2 inhibitors, a meta-analysis of randomized controlled trials (RCT) on canagliflozin reported a 32% increase in fracture risk compared to placebo or active treatment (8), and a propensity-score matched cohort study found an initial increase in fracture risk in new users of SGLT2 inhibitors compared to dipeptidyl peptidase 4 (DPP-4) inhibitors, although this effect was attenuated with longer treatment duration (9). However, most studies found neutral effects on fracture risk (10–12), including a pooled analysis of RCT data by Kohler et al. (13), a pooled analysis of RCTs by Jabbour et al. (14) and a network meta-analysis of RCTs by Tang et al. (15). GLP-1 receptor agonists have been shown to exhibit neutral effects on fracture risk in cohort studies (16, 17) and meta-analyses (18, 19), although the RCTs analyzed suffer from median follow-up durations of no more than two years (and down to 12 weeks). A recent network meta-analysis of 117 RCTs contained estimates of the risk ratios of four separate GLP-1 receptor agonists compared to four separate SGLT2 inhibitors; all but one of the 16 comparisons were statistically non-significant (20).

In the present study, we aimed to investigate fracture risk in patients using SGLT2 inhibitors versus patients using GLP-1 receptor agonists. We hypothesized no difference in fracture risk between people with T2D treated with either drug class.

## Study design and methods

The STROBE guideline for reporting of observational studies was followed (STROBE checklist can be found in **Supplemental Table S1**) (21).

### Study design and setting

We conducted a nationwide registry-based cohort study using data from the Danish national registries. We included all individuals who initiated a combination of metformin and SGLT2 inhibitor or GLP-1 receptor agonist treatment between January 1^st^ 2012 and December 31^st^ 2018. We chose to collect data from 2012 onwards as SGLT2 inhibitors became available in Denmark in 2012. Outcome information was collected by identifying all fracture-related diagnoses from index data onwards. Users of SGLT2 inhibitors were considered the exposure group, and controls (GLP-1 receptor agonist users) were matched 1:1 using propensity scores.

### Data sources

All data were provided in anonymized form by Statistics Denmark (*Danmarks Statistik*, project identifier no. 703382). Statistics Denmark obtained data from national Danish registries. All Danish citizens are assigned a unique 10-digit personal identification number (PIN) stored in the Danish Civil Registration System, which contains high-fidelity individual-level information on all residents in Denmark and Greenland (22). This PIN allows easy and unambiguous individual-level record linkage between different Danish registers (23, 24). The Danish Government provides full health care to all Danish citizens, including free access to hospitals and full or partial reimbursement of drug expenses. The Danish National Prescription Registry contains information on all prescription drugs sold in Denmark since 1995 according to the Anatomical Therapeutical Chemical (ATC) classification (25, 26). All diagnosis codes are stored in the Danish National Patient Registry, which covers all in- and outpatient contacts to the hospital (27) All physician-assigned discharge diagnoses are included, coded according to the *International Classification of Diseases, Eight Edition* (ICD-8) from 1977 until 1993 and according to ICD-10 from 1994 onwards.

All data on sex, date of birth, death, emigration, and socioeconomic factors were obtained from the Danish Civil Registration System.

### Study population

**Figure 1:**
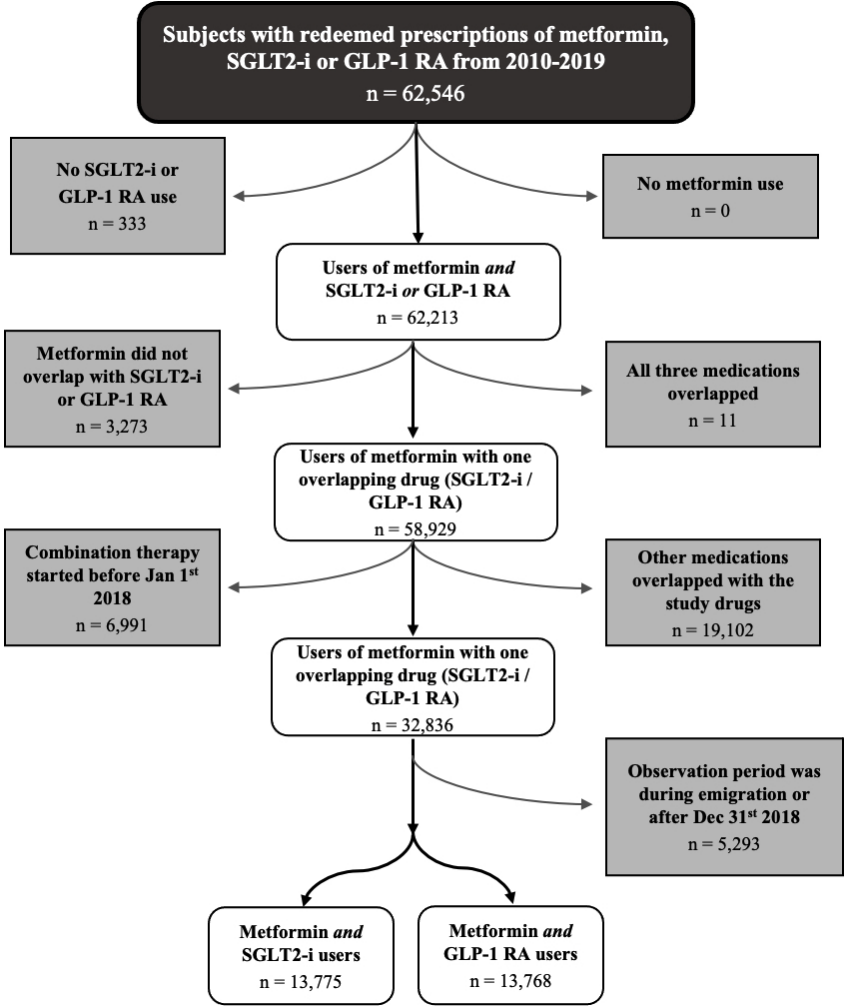
Flowchart of the process of in-/exclusion. SGLT2-i, Sodium-glucose co-transporter 2 inhibitor; GLP-1 RA, glucagon-like peptide-1 receptor agonists.

The study population included subjects alive and residing in Denmark. A flowchart of the inclusion process is presented in **Figure 1**.

The criteria for inclusion were treatment with metformin in combination with either SGLT2 inhibitors or GLP-1 receptor agonists and no concurrent treatment with any other glucose-lowering drugs between January 1^st^ 2012 and December 31^st^ 2018.

We first identified persons treated with metformin and SGLT2 inhibitors (the exposure drug) and/or GLP-1 receptor agonists (the control drug) between January 1^st^ 2010 and December 31^st^ 2019. For each medication, we defined a start date (date of first redemption) and an end date (date of last redemption plus the number of daily doses redeemed on that date). We then excluded all individuals in which treatment with SGLT2 inhibitor and GLP-1 receptor agonist overlapped for the entire duration of treatment and those in which neither medication overlapped with metformin use. Remaining individuals were assigned to the exposure or control group based on which medication was first taken singularly in combination with metformin.

Then start and end dates were defined for each other class of glucose-lowering medication. Those who were already treated with an additional glucose-lowering drug (or several) at the beginning of combination therapy were included if (and when) the third medication was halted. *End of combination therapy* was defined as the day that treatment with metformin, the exposure drug, or the control drug ceased, or when another glucose-lowering medication was initiated. Glucose-lowering drugs were defined as any medications with ATC-codes beginning in “A10”; i.e., biguanides, SGLT2-i, GLP-1 RA, DPP-4 inhibitors, insulins, sulfonylureas, alpha-glucosidase inhibitors, glitazones, and repaglinide.

Finally, the cohort was limited to those in which *beginning of combination therapy* was on or after January 1^st^ 2012.

### Exposure

The National Prescription Registry contains data on redeemed drug prescriptions along with dates, doses and pack sizes. Each medication – including the exposure and control medications – was only considered used if an individual had redeemed at least three prescriptions in the period outlined above. Medications were identified using ATC codes (**Supplemental Table S2**).

From the National Prescription Registry, we obtained the Defined Daily Dose (DDD) variable, which is “the assumed average maintenance dose per day for a drug used for its main indication in adults”, according to the World Health Organization Collaborating Centre for Drug Statistics Methodology (28). This date was added to the date of last prescription redemption to estimate a true end-of-treatment for each drug.

Of note, exposure to metformin, the exposure drug, and the control drug was in the main analysis assumed to be continuous between the dates of the first prescription redemption and end-of-treatment. To estimate the effects of pauses in these drugs, we calculated the cumulative dose (total number of DDDs) for each drug between the last prescription redeemed prior to or at index date until end of follow-up for each individual. We then assessed their compliance using the medication possession ratio (MPR); the ratio of the cumulative dose to the number of days in the same period. Individuals with an MPR < 0.5 were marked as having had a pause in the study period.

The follow-up period was defined as the time between the index date and *end of combination therapy*, emigration, death, or December 31^st^ 2018, whichever came first.

### Outcomes

The primary outcome in the study was incident major osteoporotic fractures (MOF). MOF were defined as any of the following fractures: Hip, vertebral, humerus, or forearm fracture. Fractures were identified by ICD-10 codes (**Supplemental Table S3**). Secondary analyses were performed to investigate separately the risks of any fracture, hip fracture, vertebral fracture, humerus fracture, and forearm fracture.

### Covariates

Data on covariates were obtained using ICD-8 (1977–1993) and ICD-10 (1993–2018) codes (**Supplemental Table S2**), ATC codes (1995-2018) (**Supplemental Table S3**), or a combination of both (**Supplemental Table S4**). All covariates were assessed at baseline (index date) and did not vary over time.

Age at baseline was calculated from the index date and date of birth.

Debut of diabetes was estimated as first-ever prescription for glucose-lowering drug, and diabetes duration at baseline was calculated as the time from diabetes debut until index date.

Osteoporosis was defined as the presence of diagnosis codes for osteoporosis, previous/current treatment with antiosteoporotic medications and/or previous MOF; the variable was assigned three levels (2 = previous MOF, 1 = treatment/diagnosis, 0 = none).

Previous falls were identified from diagnosis codes related to falling.

Obesity (binary variable) was identified by diagnosis codes for obesity or previous use of weight-loss medications.

Alcohol abuse (binary variable) was defined as the presence of at least one diagnosis code related to alcohol consumption (e.g., intoxication, alcoholic liver disease, alcoholic cardiomyopathy, alcohol-related psychiatric illness etc.) or previous use of medication for alcohol abstinence.

As a proxy for smoking (binary variable), we used diagnosis codes related to lung diseases highly associated with tobacco exposure along with diagnosis codes for nicotine poisoning and psychiatric tobacco-related diagnoses. In addition, previous use of medications for the treatment of tobacco dependence and initiation of drugs for obstructive airway disease after the age of 40 were used as proxies for smoking. We expect this variable to represent heavy smoking.

Hypertension was defined by any diagnosis code for hypertension and/or ever use of an antihypertensive agent.

Hyperthyroidism was identified through diagnosis codes or treatment with any antithyroid medication.

Diabetic nephropathy, diabetic retinopathy, diabetic neuropathy, inflammatory bowel disease (IBD), kidney disease, chronic pancreatitis, visual impairment, hyperparathyroidism, and eating disorder/malabsorption were identified through diagnosis codes.

Previous insulin use and previous glucocorticoid use were identified through redeemed prescriptions.

The Charlson Comorbidity Index (CCI) was calculated based on other comorbidities. The CCI was modified to exclude kidney disease and late-diabetic complications, as these covariates were separately adjusted for in the statistical analyses.

Income, marital status and employment status (classified by Statistics Denmark according to the so-called *SOCIO13 classification*) were identified on the year preceding each individual’s index year. Income (in Danish Kroner, DKK) was adjusted for inflation to a 2018 level according to the Consumer Price Index provided by Statistics Denmark and converted from DKK to Euros using an exchange rate of 7.4363 DKK/Euro..

### Statistical analysis

#### Descriptive statistics

Descriptive statistics are presented as numbers and proportions (%), means and standard deviations (SD), or medians and interquartile ranges (IQR). Standardized mean differences (SMD) were also calculated for all baseline variables as recommended for propensity-score matched studies (29). Cohen suggested that SMD values above 0.2 be considered small, SMD values above 0.5 considered medium-sized, and SMD values above 0.8 considered large (29, 30).

#### Missing data

There were only missing data in the socioeconomic variables (marital status, income, and employment). Income was used as a covariate in the main analysis, and missing data were imputed beforehand. Missing data were assumed to be missing at random, and multivariate imputation by chained equations, a method of performing multiple imputations, was performed (31, 32). Ten imputations were produced, each of which ran for ten iterations. As the proportion of missing data was very low (0.2%), and the covariate (income) appeared to be balanced between groups and not alter the results of the survival analysis, it – and imputation – was omitted from all subgroup and sensitivity analyses.

#### Propensity-score matching

Due to imbalances in sex, age at baseline, and inclusion date (with GLP-1 receptor agonists having been introduced in Denmark approximately 5 years before SGLT2 inhibitors), we opted to match the two groups on propensity scores estimated from these variables. To produce these, we fitted a binomial logistic model to age, sex, and (a numeric value for) the inclusion date with treatment group as the dependent variable (33, 34). From the logistic regression, we predicted propensity scores for each individual in the main cohort.

To minimize bias, we matched subjects on the logit transformation of the propensity score using nearest-neighbor (“greedy”) matching without replacement, using a caliper width equal to 0.2 x the SD of the transformed propensity scores (35, 36). As homogeneity of variances was violated (variance ratio of 2.5 between groups), the variance of the control group was used to set the caliper width.

For multiple imputed datasets, matching and statistical analysis were performed separately on each resultant dataset, and the statistical estimates were finally pooled.

After matching, balance in the matched variables was assessed by inspecting the distributions of propensity scores across groups and by calculating SMDs for each variable.

#### Multicollinearity

Multicollinearity was assessed using the Variance Inflation Factor (VIF) which yielded values no higher than 1.4 for any covariate. In addition, we examined Pearson’s partial correlation coefficient for each pair of variables, and none revealed significant correlations.

#### Survival analysis

On a non-imputed matched dataset, the Kaplan-Meier Estimator was used to produce survival plots for all outcomes; a survival plot for MOF on a non-matched dataset was also produced (37).

For the primary analysis, we used the Cox proportional hazards model to estimate hazard rate ratios (HRs) for fracture between the exposure and the control groups. We estimated both crude and adjusted HRs for primary and secondary outcomes. The proportional hazards assumption was evaluated by examining the scaled Schoenfeld residuals of the Cox model and finding no trend with time for any variable (38). To account for pairing in the matched dataset, stratification by matched pairs or a robust variance estimator can be utilized (39, 40); as stratification may result in biased estimation of marginal hazard ratios, a robust variance estimator was used.

Finally, to also allow a non-multiplicative effect of SGLT2 inhibitors on fracture risk, we used Aalen’s additive regression model to examine whether absolute rather than relative differences in hazard existed between the groups (41).

### Sensitivity and subgroup analyses

We performed several sensitivity and subgroup analyses. For each subgroup, we performed matching anew using the previously computed propensity scores.

First, we split our cohort into males and females. Second, we performed an analysis excluding all who had pauses (MPR < 0.5) in their metformin or study drug (SGLT2 inhibitor or GLP-1 receptor agonist) during the study period. Third, we examined whether excluding individuals with kidney disease, previous pancreatitis, and previous falls would affect the results. Fourth, we examined whether excluding individuals with short follow-up time (less than 6 months) – who had not had enough time to manifest potential fractures – led to a difference in fracture risk. Fifth, due to previous studies hinting at possible drug-differential effects, we split the SGLT2 inhibitor group into specific drug groups based on which specific drug – canagliflozin, empagliflozin, or dapagliflozin – they had received the largest cumulative dose of during the study period. Ties were handled by allowing a person to appear in several of these subgroups; only three persons did so. Sixth, we examined the full cohort without matching. Seventh, we treated glucocorticoids as a reason for exclusion. Treatment with systemic glucocorticoids within the last year prior to inclusion was not allowed, and follow-up did not continue past initiation of systemic glucocorticoids. Lastly, we performed an analysis more similar to the “intention-to-treat” approach in clinical trials, in which we continued follow-up after changes in medication for an extra 2 years – or until death or emigration, whichever came first. This was to examine possible slow-emerging and/or long-lasting effects of the exposure on fracture risk.

#### Statistical software

All analyses were performed using R 4.1.0 (The R Core Team & The R Foundation for Statistical Computing, Vienna, Austria) in the integrated development environment (IDE) RStudio 1.4.1106 (RStudio, PBC, Boston, MA, USA). For imputation, the package “mice” (v 3.13.0) was used. Matching was performed using “MatchIt” (v. 4.2.0) and, for multiply imputed datasets, “MatchThem” (v. 1.0.0). Survival analyses – i.e., Cox model, Kaplan-Meier estimator, and Aalen’s additive regression model – were performed using packages “Survival” (v. 2.1.11), “Survminer” (v. 0.4.9), and “Survey” (v. 4.0).

## Results

### Baseline characteristics

We identified 27,543 subjects treated with metformin in combination with either SGLT2 inhibitors (n = 13,775) or GLP-1 receptor agonists (n = 13,768). After propensity-score matching, a total of 18,380 (9,190 in each group) remained.

Matching was satisfactory, although due to the large effects of inclusion date and sex, the difference in age was not reduced.

**Table 1** shows baseline characteristics of subjects in either group in both the full cohort and the matched cohort. Data from the matched cohort will be presented in short.

**Table 1:** Baseline Characteristics of Full and Matched Cohorts.

	Full Cohort		Matched Cohort		
	SGLT2-i group	GLP-1 RA group	SGLT2-i group	GLP-1 RA group	SMD
	13,775	13,768	9,190	9,190	
**Sex (female),** n (%)	4,934 (35.8%)	5,840 (42.4%)	3,540 (38.5%)	3,680 (40.0%)	0.031
**Age (years),** mean (±SD)	60.0 (±11.4)	57.4 (±12.1)	61.1 (±11.3)	58.5 (±12.0)	**0.218**
**Follow-up (days),** median [IQR]	334 [139–662]	497 [185–1,077]	355 [126–779.8]	372 [136.2–766]	0.011
**Inclusion Year,** n (%)					0.179
2012	4 (0.0%)	2,482 (18.0%)	4 (0.0%)	61 (0.7%)	
2013	394 (2.9%)	1,841 (13.4%)	394 (4.3%)	329 (3.6%)	
2014	664 (4.8%)	1,544 (11.2%)	664 (7.2%)	957 (10.4%)	
2015	1179 (8.6%)	1,767 (12.8%)	1,156 (12.6%)	1,709 (18.6%)	
2016	2,494 (18.1%)	1,776 (12.9%)	1,823 (19.8%)	1,776 (19.3%)	
2017	3,780 (27.4%)	1,885 (13.7%)	1,916 (20.8%)	1,885 (20.5%)	
2018	5,260 (38.2%)	2,473 (18.0%)	3,233 (35.2%)	2,473 (26.9%)	
**Diabetes Duration (years),** median [IQR]	5.80 [2.62–9.14]	5.56 [2.57–9.20]	5.96 [2.80–9.35]	5.91 [2.80–9.61]	0.024
**Charlson Comorbidity Index,** mean (±SD)	0.73 (±1.17)	0.72 (±1.14)	0.76 (±1.19)	0.79 (±1.19)	0.022
**Complications of diabetes,** n (%)	2,472 (17.9%)	3,557 (25.8%)	1,687 (18.4%)	2,325 (25.3%)	0.169
Diabetic Neuropathy	385 (3.8%)	563 (4.1%)	268 (2.9%)	378 (4.1%)	0.065
Diabetic Nephropathy	213 (1.5%)	450 (3.3%)	141 (1.5%)	319 (3.5%)	0.124
Diabetic Retinopathy	709 (5.1%)	915 (6.6%)	498 (5.4%)	558 (6.1%)	0.028
Other	1,642 (11.9%)	2,477 (18.0%)	1,116 (12.1%)	1,631 (17.7%)	0.158
**Osteoporosis,** n (%)					0.030
No history	12,126 (88.0%)	12,167 (88.4%)	8,090 (88.0%)	8,073 (87.8%)	
Diagnosed / Treated	273 (2.0%)	212 (1.5%)	183 (2.0%)	146 (1.6%)	
Previous MOF	1,376 (10.0%)	1,389 (10.1%)	917 (10.0%)	971 (10.6%)	
**Risk factors for falls,** n (%)					
Hypoglycemic episodes	94 (0.7%)	115 (0.8%)	66 (0.7%)	86 (0.9%)	0.024
Previous Falls	516 (3.7%)	575 (4.2%)	353 (3.8%)	405 (4.4%)	0.028
Visual Impairment	185 (1.3%)	153 (1.1%)	131 (1.4%)	106 (1.2%)	0.024
**Any pancreatitis,** n (%)	313 (2.3%)	226 (1.6%)	211 (2.3%)	145 (1.6%)	0.052
Acute Pancreatitis	267 (1.9%)	210 (1.5%)	181 (2.0%)	133 (1.4%)	0.040
Chronic Pancreatitis	97 (0.7%)	38 (0.3%)	72 (0.8%)	24 (0.3%)	0.073
**Glucose-Lowering Drugs,** n (%)					
Metformin	13,561 (98.4%)	13,527 (98.2%)	9,069 (98.7%)	9,025 (98.2%)	0.039
SGLT2 inhibitors	1,782 (12.9%)	493 (3.6%)	1,205 (13.1%)	483 (5.3%)	**0.275**
GLP-1 receptor agonists	261 (1.9%)	4,447 (32.3%)	178 (1.9%)	2,904 (31.6%)	**0.865**
DDP4 inhibitors	2,347 (17.0%)	3,336 (24.2%)	1,612 (17.5%)	2,408 (26.2%)	**0.211**
Insulin, any	890 (6.5%)	1,772 (12.9%)	582 (6.3%)	1,220 (13.3%)	**0.235**
Sulfonylureas	3,572 (25.9%)	5,030 (36.5%)	2,557 (27.8%)	3,066 (33.4%)	0.120
Alpha-glucosidase inhibitors	32 (0.2%)	92 (0.7%)	24 (0.3%)	63 (0.7%)	0.062
Glitazones	284 (2.1%)	525 (3.8%)	218 (2.4%)	269 (2.9%)	0.035
Repaglinide	125 (0.9%)	185 (1.3%)	87 (0.9%)	104 (1.1%)	0.018
**Hypertension,** n (%)	10,818 (78.5%)	11,080 (80.5%)	7,327 (79.7%)	7,461 (81.2%)	0.037
**Chronic Kidney Disease,** n (%)	321 (2.3%)	499 (3.6%)	218 (2.4%)	399 (4.3%)	0.110
**Liver Disease,** n (%)	433 (3.1%)	409 (3.0%)	289 (3.1%)	294 (3.2%)	0.003
Mild	390 (2.8%)	382 (2.8%)	259 (2.8%)	278 (3.0%)	0.012
Moderate to severe	84 (0.6%)	64 (0.5%)	54 (0.6%)	44 (0.5%)	0.015
**Hyperparathyroidism,** n (%)	54 (0.4%)	82 (0.6%)	42 (0.5%)	62 (0.7%)	0.029
**Hyperthyroidism,** n (%)	364 (2.6%)	386 (2.8%)	271 (2.9%)	248 (2.7%)	0.015
**Hypogonadism,** n (%)	24 (0.2%)	39 (0.3%)	15 (0.2%)	32 (0.3%)	0.037
**Eating disorder or malabsorption,** n (%)	98 (0.7%)	83 (0.6%)	66 (0.7%)	62 (0.7%)	0.004
**Venous thromboembolism,** n (%)	1,014 (7.4%)	1,144 (8.3%)	723 (7.9%)	792 (8.6%)	0.027
**Inflammatory bowel disease,** n (%)	450 (3.3%)	480 (3.5%)	311 (3.4%)	346 (3.8%)	0.021
**Osteoarthritis,** n (%)	2,261 (16.4%)	2,445 (17.8%)	1,614 (17.6%)	1,745 (19.0%)	0.037
**Dementia,** n (%)	808 (5.9%)	801 (5.8%)	560 (6.1%)	588 (6.4%)	0.013
**Alcohol abuse,** n (%)	1,012 (7.3%)	1,000 (7.3%)	678 (7.4%)	680 (7.4%)	0.001
**Smoking,** n (%)	4,266 (31.0%)	4,627 (33.6%)	2,921 (31.8%)	3,190 (34.7%)	0.062
**Obesity,** n (%)	3,509 (25.5%)	5,373 (39.0%)	2,434 (26.5%)	3,420 (37.2%)	**0.232**
**Other medications,** n (%)					
Statins	11,214 (81.4%)	11,136 (80.9%)	7,551 (82.2%)	7,479 (81.4%)	0.020
Thiazides	5,080 (36.9%)	5,889 (42.8%)	3,551 (38.6%)	3,973 (43.2%)	0.093
Loop Diuretics	2,655 (19.3%)	3,530 (25.6%)	1,925 (20.9%)	2,416 (26.3%)	0.126
Potassium-sparing diuretics	1,428 (10.4%)	1,716 (12.5%)	1,003 (10.9%)	1,193 (13.0%)	0.064
Antipsychotic drugs	1,730 (12.6%)	1,770 (12.9%)	1,125 (12.2%)	1,152 (12.5%)	0.009
Antiepileptic drugs	2,003 (14.5%)	2,231 (16.2%)	1,329 (14.5%)	1,596 (17.4%)	0.079
Antiarrhythmic drugs	214 (1.6%)	235 (1.7%)	147 (1.6%)	177 (1.9%)	0.025
Hypnotics	3,876 (28.1%)	4,158 (30.2%)	2,680 (29.2%)	2,818 (30,7%)	0.033
Antidepressants	4,691 (34.1%)	5,320 (38.6%)	3,123 (34.0%)	3,559 (38.7%)	0.099
Anxiolytics	3,645 (26.5%)	3,996 (29.0%)	2,501 (27.2%)	2,644 (28.8%)	0.035
Opioids	7,799 (56.6%)	8,199 (59.6%)	5,246 (57.1%)	5,561 (61.5%)	0.090
NSAID	12,144 (88.2%)	12,344 (89.7%)	8,138 (88.6%)	8,289 (90.2%)	0.053
Sex hormones	3,425 (24.9%)	4,333 (31.5%)	2,447 (26.6%)	2,792 (30.4%)	0.083
Antacids	7,378 (53.6%)	7,498 (54.5%)	5,014 (54.6%)	5,204 (56.5%)	0.042
Glucocorticoids	4,597 (33.4%)	4,736 (34.4%)	3,153 (34.3%)	3,259 (35.5%)	0.024
**Income (euros),** median [IQR]	34,109 [24,590–50,254]	34,885 [25,307–50,504]	*33,100* *[24,233–48,944]*	*34,800* *[25,188–50,482]*	*0.022*
**Income quintiles,** n (%)					*0.048*
1^st^	2,876 (20.9%)	2,622 (19.0%)	*1,972 (21.5%)*	*1,792 (19.5%)*	
2^nd^	2,697 (19.6%)	2,802 (20.4%)	*1,910 (20.8%)*	*1,796 (19.5%)*	
3^rd^	2,724 (19.8%)	2,774 (20.1%)	*1,823 (19.8%)*	*1,901 (20.7%)*	
4^th^	2,696 (19.6%)	2,803 (20.4%)	*1,774 (19.3%)*	*1,856 (20.2%)*	
5^th^	2,755 (20.0%)	2,744 (19.9%)	*1,698 (18.5%)*	*1,827 (19.9%)*	
Missing Data	27 (0.2%)	23 (0.2%)	*13 (0.1%)*	*18 (0.2%)*	
**Marital Status,** n (%)					0.073
Unmarried	2,501 (18.2%)	2,723 (19.8%)	1,530 (16.6%)	1,785 (19.4%)	
Married / Registered Partnership	7,920 (57.5%)	7,831 (56.9%)	5,356 (58.3%)	5,166 (56.2%)	
Divorced / Annulled Partnership	2,265 (16.4%)	2,264 (16.4%)	1,492 (16.2%)	1,559 (17.0%)	
Widowed	1,035 (7.5%)	899 (6.5%)	783 (8.5%)	641 (7.0%)	
Missing Data	54 (0.4%)	51 (0.4%)	29 (0.3%)	39 (0.4%)	
**SOCIO13 group,** n (%)					0.088
Working	6,039 (43.8%)	6,235 (45.3%)	3,799 (41.3%)	4,041 (44.0%)	
Unemployed	1,186 (8.5%)	1,249 (9.1%)	704 (7.7%)	816 (8.9%)	
Retired	6,182 (44.9%)	5,879 (42.7%)	4,469 (48.6%)	4,066 (44.2%)	
Student	40 (0.3%)	112 (0.8%)	23 (0.3%)	72 (0.8%)	
Other	301 (2.2%)	270 (2.0%)	182 (2.0%)	177 (1.9%)	
Missing Data	54 (0.4%)	51 (0.4%)	13 (0.1%)	18 (0.2%)	

Alle data are presented as n (%), mean (±SD), or median [IQR]. SGLT2-i, sodium-glucose cotransporter 2 inhibitor; GLP-1 RA, glucagon-like peptide-1 receptor agonists; SMD, standardized mean difference. SMDs above 0.2 are highlighted with bold font. Data on income in the matched cohort (italicized) are presented without imputations.

Follow-up time was balanced between the two groups with a median [IQR] of 355 [126–780] days in the SGLT2 inhibitor group and 373 [136–766] days in the control group. In total, we had 25,586 years of combined follow-up time.

Subjects in the SGLT2 inhibitor group were less likely to be female (38.5% vs. 40.0%) and were slightly older with mean ( ± SD) age of 61.1 ( ± 11.3) vs. 58.5 ( ± 12.0) years in the GLP-1 receptor agonist control group. Median [IQR] diabetes durations in the SGLT2 inhibitor group was 5.96 [2.80–9.35] years and, similarly, 5.91 [2.80–9.61] in the controls, and mean ( ± SD) CCI scores were 0.76 ( ± 1.19) and 0.79 ( ± 1.19) in the SGLT2 inhibitor and control group, respectively. Previous MOF were equally prevalent in both groups (10.0% vs. 10.6% in the SGLT2 inhibitor and control group, respectively).

Subjects in the control group had more complications of diabetes (25.3% vs. 18.4%), a lower occurrence pancreatitis (1.6% vs. 2.3%), and a higher prevalence of chronic kidney disease (4.3% vs. 2.4%), although all these effects sizes were below the minimum SMD threshold of 0.2. In addition, those in the control group were more likely to have a history of obesity (37.2% vs. 26.5%, SMD 0.232). In addition, the SGLT2 inhibitor group had a slightly larger fraction of subjects included in 2018, and a smaller fraction included in the years 2012, 2014, and 2015. The only covariates with SMDs above the threshold of 0.2 (for small differences) were age, obesity, and previous use of insulins, SGLT-2 inhibitors, DPP-IV inhibitors, and GLP-1 receptor agonists; with GLP-1 receptor agonists exhibiting by far the largest difference (SMD 0.865).

Socioeconomic variables were balanced between groups.

### Risk of major osteoporotic fractures

**Figure 2:**
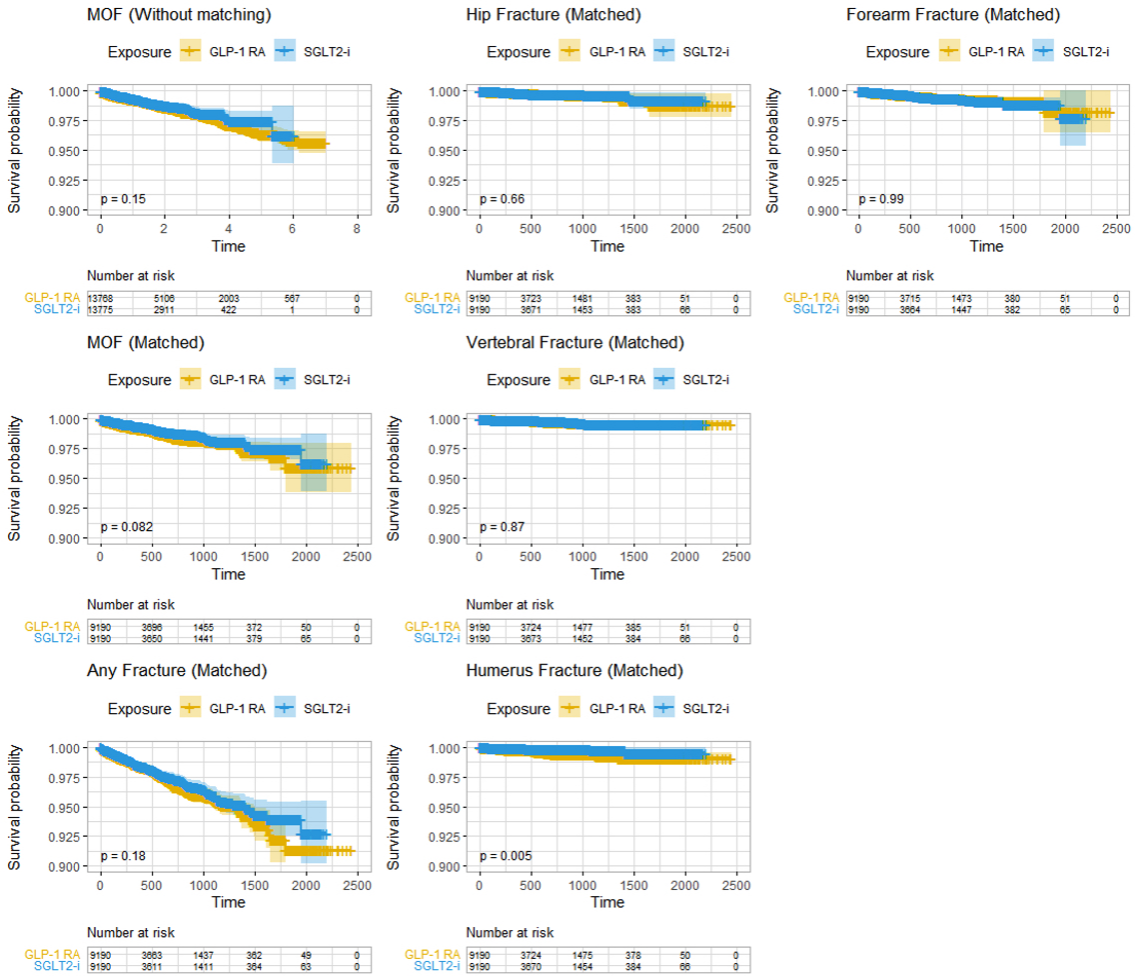
Kaplan-Meier Survival Curves of fracture. Survival curves are presented with *number-at-risk* tables. Time in days on the x-axes. Note, the y-axes go from 0.90 to 1.00. MOF, Major osteoporotic fracture; GLP-1 RA, Glucagon-like peptide-1 receptor agonists; SGLT2-i, sodium-glucose cotransporter 2 inhibitors.

**Table 2** presents HRs for fractures in the matched cohort during the study period. A MOF occurred in 0.8% (n = 74) and 1.1% (n = 97) of SGLT2 inhibitor users and GLP-1 receptor agonist users, respectively. The Crude HR for MOF in the SGLT2 inhibitor group was 0.77 [0.57–1.04]. When adjusted for age and sex, this became statistically significant (HR 0.73 [0.54–0.99], although the effect was attenuated again in the fully adjusted model (HR 0.77 [0.56–1.05]). For each analysis in **Table 2** and for the unmatched analysis of MOF, we also present Kaplan-Meier survival curves for crude illustrations (**Figure 2**), which similarly yielded non-significant results.

**Table 2:** Hazard Ratios (HR) for various fracture types in the matched cohort.

Fracture	Fractures, n (%)	Unadjusted (HR [95% CI])	Age, Sex-HR [95% CI]	Full Model-HR [95% CI]
**MOF**	SGLT2-i: 74 (0.8)	0.77 [0.57 – 1.04]	**0.73 [0.54 – 0.99]**	Model 1: 0.77 [0.56 – 1.05]
	GLP-1 RA: 97 (1.1)			
**Any**	SGLT2-i: 174 (1.9)	0.87 [0.71 – 1.07]	0.86 [0.70 – 1.05]	Model 1: 0.91 [0.74 – 1.12]
	GLP-1 RA: 201 (2.2)			
**Hip**	SGLT2-i: 19 (0.2)	0.87 [0.47 – 1.61]	0.80 [0.43 – 1.49]	Model 2: 0.87 [0.45 – 1.67]
	GLP-1 RA: 22 (0.2)			
**Vertebral**	SGLT2-i: 14 (0.2)	0.94 [0.45 – 1.95]	0.88 [0.43 – 1.83]	Model 2: 0.86 [0.40 – 1.88]
	GLP-1 RA: 15 (0.2)			
**Humerus**	SGLT2-i: 11 (0.1)	**0.38 [0.20 – 0.76]**	**0.36 [0.18 – 0.71]**	Model 2: **0.35 [0.18 – 0.70]**
	GLP-1 RA: 29 (0.3)			
**Forearm**	SGLT2-i: 35 (0.4)	1.00 [0.63 – 1.60]	1.00 [0.62 – 1.59]	Model 2: 1.14 [0.70 – 1.86]
	GLP-1 RA: 35 (0.4)			

HR, Hazard Ratio; MOF, major osteoporotic fracture; SGLT2-i, sodium-glucose cotransporter 2 inhibitor; GLP-1 RA, glucagon-like peptide-1 receptor agonists; Bold font: the HR was significantly different from 1.00.

Full model 1: Adjusted for sex, age, inclusion date, diabetes duration, Charlson Comorbidity Index, diabetic nephropathy, diabetic retinopathy, diabetic neuropathy, previous falls, inflammatory bowel disease, previous insulin use, previous glucocorticoid use, osteoporosis (including prevalent MOF), hypertension, kidney disease, alcohol, smoking, obesity, income, chronic pancreatitis, visual impairment, hyperthyroidism, hyperparathyroidism, eating disorder/malabsorption.

Full model 2: Corresponding to Model 1 but excluding chronic pancreatitis, diabetic neuropathy, visual impairment, hyperthyroidism, hyperparathyroidism and eating disorder/malabsorption as covariates.

The Crude HR for any fracture was 0.87 [0.71–1.07], and the fully adjusted HR was 0.91 [0.74-1.12].

Examining HRs for each specific type of MOF yielded generally similar results. The crude HR for hip fracture was 0.87 [0.47–1.61], which was unaltered in the fully adjusted model (HR 0.87 [0.45–1.67]). The crude HR for vertebral fractures was 0.94 [0.45–1.95] with negligible change after full adjustment (HR 0.86 [0.40–1.88]). For forearm, the crude HR was 1.00 [0.63–1.60] and the fully adjusted HR 1.14 [0.70–1.86]. In contrast, the analysis of humerus fractures indicated a protective effect with an adjusted HR of 0.35 [0.18–0.70]. However, there were very few events for each subtype of fracture, making interpretation difficult.

### Subgroup and sensitivity analyses

Various subgroup and sensitivity analyses yielded similarly non-significant results (**Table 3**).

**Table 3:** Hazard Ratios for MOF in subgroup and sensitivity analyses.

Analysis	n =	Fractures, n (%)	Unadjusted (HR [95% CI])	Age, (Sex)-HR [95% CI]	Full Model-HR [95% CI]
**Males**	SGLT2-i: 5,377	30 (0.6)	0.75 [0.47 – 1.20]	0.74 [0.46 – 1.19]	Model 2 0.80 [0.50 – 1.29]
	GLP-1 RA: 5,377	38 (0.7)			
**Females**	SGLT2-i: 3,795	50 (1.3)	0.87 [0.60 – 1.26]	0.80 [0.55 – 1.16]	Model 2 0.83 [0.56 – 1.22]
	GLP-1 RA: 3,795	63 (1.7)			
**No Pause**	SGLT2-i: 7,432	65 (0.9)	0.84 [0.60 – 1.17]	0.79 [0.56 – 1.10]	Model 2 0.84 [0.60 – 1.18]
	GLP-1 RA: 7,432	78 (1.0)			
**No CKD etc.**	SGLT2-i: 8,309	60 (0.7)	0.73 [0.52 – 1.02]	0.73 [0.52 – 1.02]	Model 2 0.80 [0.56 – 1.13]
	GLP-1 RA: 8,309	82 (1.0)			
**6+ months follow-up**	SGLT2-i: 6,458	63 (1.0)	0.73 [0.53 – 1.01]	**0.72 [0.52 – 0.99]**	Model 2 0.77 [0.55 – 1.07]
	GLP-1 RA: 6,458	89 (1.4)			
**Canagliflozin**	SGLT2-i: 302	1 (0.3)	0.42 [0.11 – 1.53]	0.42 [0.10 – 1.69]	N/A
	GLP-1 RA: 302	2 (0.7)			
**Empagliflozin**	SGLT2-i: 6,893	49 (0.7)	0.78 [0.54 – 1.13]	0.77 [0.53 – 1.12]	Model 2 0.80 [0.55 – 1.17]
	GLP-1 RA: 6,893	65 (0.9)			
**Dapagliflozin**	SGLT2-i: 5,772	48 (0.8)	0.70 [0.48 – 1.02]	0.70 [0.48 – 1.02]	Model 2 0.81 [0.55 – 1.19]
	GLP-1 RA: 5,772	60 (1.0)			
**Full cohort (unmatched)**	SGLT2-i: 13,775	105 (0.8)	0.84 [0.66 – 1.07]	0.82 [0.64 – 1.05]	Model 1 0.78 [0.59 – 1.03]
	GLP-1 RA: 13,768	189 (1.4)			
**Glucocorticoid as exclusion**	SGLT2-i: 8,464	62 (0.7)	0.74 [0.54 – 1.03]	**0.70 [0.50 – 0.97]**	Model 1 0.73 [0.52 – 1.03]
	GLP-1 RA: 8,464	84 (1.0)			
**Intention-to-treat analysis**	SGLT2-i: 9,190	116 (1.3)	0.95 [0.74 – 1.22]	0.87 [0.68 – 1.12]	Model 1 0.94 [0.72 – 1.21]
	GLP-1 RA: 9,190	135 (1.5)			
**Age: <65**	SGLT2-i: 6,088	37 (0.6)	0.73 [0.48 – 1.12]	0.72 [0.47 – 1.10]	Model 2 0.81 [0.51 – 1.28]
	GLP-1 RA: 6,088	50 (0.8)			
**Age: 65–74**	SGLT2-i: 2,401	26 (1.1)	0.89 [0.53 – 1.49]	0.87 [0.52 – 1.47]	Model 2 1.02 [0.59 – 1.77]
	GLP-1 RA: 2,401	31 (1.3)			
**Age: ≥ 75**	SGLT2-i: 670	11 (1.6)	0.60 [0.29 – 1.23]	0.54 [0.27 – 1.09]	N/A
	GLP-1 RA: 670	19 (2.8)			

HR, Hazard Ratio; MOF, major osteoporotic fracture; SGLT2-i, sodium-glucose cotransporter 2 inhibitor; GLP-1 RA, glucagon-like peptide-1 receptor agonists; Bold font = the HR was significantly different from 1.00.

“No pause”: excluded those with pauses in metformin, SGLT2 inhibitor or GLP-1 receptor agonist during the study period. “No CKD etc.”: Excluded those with chronic kidney disease, previous falls and previous chronic pancreatitis. “6+ months follow-up”: Excluding all with follow-up times less than 183 days.

Full model 1: Adjusted for sex, age, inclusion date, diabetes duration, Charlson Comorbidity Index, diabetic nephropathy, diabetic retinopathy, diabetic neuropathy, previous falls, inflammatory bowel disease, previous insulin use, previous glucocorticoid use, osteoporosis (including prevalent MOF), hypertension, kidney disease, alcohol, smoking, obesity, chronic pancreatitis, visual impairment, hyperthyroidism, hyperparathyroidism, eating disorder/malabsorption.

Full model 2: Corresponding to Model 1 but excluding chronic pancreatitis, diabetic neuropathy, visual impairment, hyperthyroidism, hyperparathyroidism and eating disorder/malabsorption as covariates. N/A means "Not applicable.

Effects were similar between males and females. When excluding those with pauses in medication or those with chronic kidney disease, previous pancreatitis and previous falls did not alter the results, either. When excluding subjects with follow-up times less than 6 months, 12,916 individuals remained. In this group, we found an unadjusted HR of 0.73 [0.53–1.01] which was similarly to the main analysis significant upon adjusting for age and sex but once again attenuated in the fully adjusted model (HR 0.77 [0.55–1.07]).

Dividing the SGLT2 inhibitor group into subgroups based on which specific drug yielded three groups; canagliflozin, empagliflozin, and dapagliflozin. Neither empagliflozin nor dapagliflozin showed effects different from the main results. Only 302 individuals were in the canagliflozin group, and although an unadjusted HR of 0.42 [0.11–1.53] was found, this result was based on a mere total of three fractures.

Examining the full (unmatched) cohort yielded similar results (unadjusted HR 0.84 [0.66–1.07] and fully adjusted HR 0.78 [0.59–1.03]).

Defining recent or ongoing glucocorticoid use as an exclusion criterion did not impact the results (adjusted HR 0.73 [0.52–1.03]).

In addition, performing an “intention-to-treat” analysis yielded an adjusted HR of 0.94 [0.72–1.21], slightly closer to a fully neutral effect.

**Figure 3:**
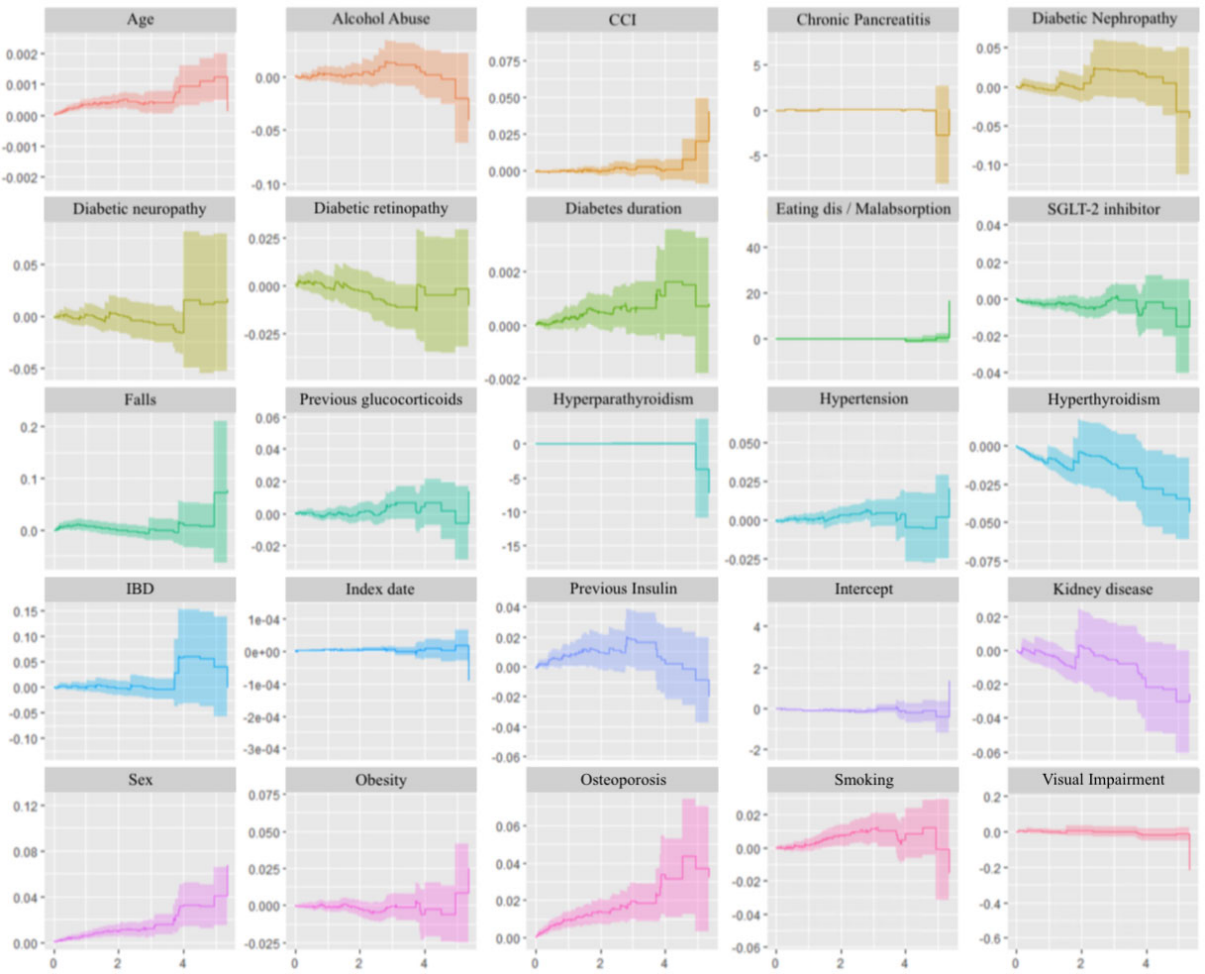
Aalen’s Additive Regression Plots. Plots of the time-varying additive hazards plotted against time (years) on the x-axis for covariates used in Aalen’s Additive Regression Model. CCI, Charlson Comorbidity Index; Eating dis, eating disorder; IBD, inflammatory bowel disease. This regression model assumes that the risks attributable to each risk factor are additive (producing hazard rate differences) rather than multiplicative (hazard rate ratios). Each plot shows the cumulative hazard associated with a given covariate at each time point – the slopes at any point in time represent hazard rates, and positive slopes correspond to increased risk, whereas negative slopes correspond to reduced risk. As all effects are allowed to be time-varying, a covariate may at one timepoint increase risk and a reduce risk at another timepoint. The intercept term represents a baseline hazard; i.e., the hazard when the contributions from all covariates (including exposure) are zero.

Finally, we performed an entirely separate test of MOF hazard on the matched cohort using the Aalen’s additive regression model (**Figure 3**). This test revealed no time-varying effects of the exposure/control drugs with a slope of -0.0058 (p = 0.08).

As a final measure, we analyzed deaths in the two groups to examine whether an imbalance in these may have influenced the results, as competing risks were not formally accounted for in the main analyses. In the SGLT2 inhibitor group, 59 (0.6%) deaths occurred with a median [IQR] time-to-event of 286 [124–828] days, whereas the GLP-1 receptor agonist group experienced 84 (0.9%) deaths with a median time-to-event of 188 [54–670] days. Indeed, the crude HR for death (with MOF as a censoring event) in the SGLT2 inhibitor group with the GLP-1 receptor agonist group as reference was 0.70 [0.51–0.98]. When adjusted for age and sex, this became 0.65 [0.47–0.91] and when fully adjusted 0.81 [0.58–1.12].

## Discussion

### Summary of findings

In the present study, we found that the risk of MOF was similar between treatment with GLP-1 receptor agonist and SGLT2 inhibitors as add-on therapies to metformin. Whereas some other research has indicated bone protective effects of GLP-1 receptor agonists and bone detrimental effects of SGLT2 inhibitors (perhaps particularly canagliflozin), our results showed a small, non-significant trend toward fewer fractures with SGLT2 inhibitors.

We found no drug-differential effects but were unfortunately unable – due to small sample size – to evaluate the risk with canagliflozin.

Examining specific fracture sites revealed no difference between SGLT2 inhibitors and GLP-1 receptor agonists in the cases of hip, forearm, and vertebral fractures. Only in the case of humerus fractures did our results reveal a statistically significant effect. However, this secondary analysis was based on only 40 fractures in total, and our study has not taken multiple testing into account, which means that significance is to be expected at some level, even if not clinically meaningful. Indeed, the authors are not aware of a mechanism whereby the drugs would have a protective effect on the humerus but not on other bone tissue.

In our sensitivity analysis in which subjects were followed for up to an additional two years, we found HRs closer to 1.00 than in the main analysis. This suggests that there are no long-term detrimental effects on bone by either drug after discontinuation, switch, or addition of other glucose-lowering drugs.

As increased fall risk may be a contributor to the fracture risk in diabetes (42), we attempted to compensate for this by performing a subgroup analysis without those with previous diagnosis codes pertaining to falls. In addition, we adjusted for covariates related to falls, diabetic neuropathy, diabetic retinopathy, and visual impairment.

We speculated whether differential mortality in the two groups may have influenced the results, and found a difference, albeit relatively small and non-significant when fully adjusted. A higher mortality in the GLP-1 receptor agonist group would mean an overestimation of fracture hazard in this group. Therefore, the true hazard ratio may be slightly closer to 1, but as deaths were so rare, it is unlikely that any such bias will have produced our results if the true hazard ratio were above 1.

### Previous research

SGLT2 inhibitors became available in Denmark in 2012 as a treatment for T2D. Most observational (10–12) and (13–15) clinical studies have found neutral effects on fracture risk with SGLT2 inhibitors, although one meta-analysis of RCTs with long follow-up found increased fracture risk in canagliflozin treatment (8). For GLP-1 receptor agonists, observational studies and meta-analyses of RCTs on fracture risk have found mostly neutral effects (16–20, 43), although one meta-analysis found reduced risk of fractures (44).

Most studies, however, are limited by short follow-up durations (7). Furthermore, interpretation of the body of observational research is generally made difficult in the context of glucose-lowering drugs by the heterogeneity inherent in the variety of study designs, particularly the choice of many different comparators. In contrast, it is rarely feasible to perform clinical studies on the timescales required for proper evaluation of such long-term outcomes as osteoporotic fractures.

### Strengths and limitations

This cohort study was performed on a nationwide level with individual-level data on all prescription medications and diagnosis codes along with a variety of socioeconomic factors. This allows access to high-fidelity information on treatments and comorbidities in the whole period in which SGLT2 inhibitors have been marketed in Denmark with limited missing data using an unbiased study population, providing results that are highly generalizable to populations at a wide range of ages that are comparable to the Danish population.

The use of GLP-1 receptor agonists as a comparator provided a highly comparable control group, particularly as both drugs were used in the setting of sole add-on medication to metformin. As both drugs have equal priority in the management of T2D, we expect very limited confounding by indication to appear in this study. However, GLP-1 receptor agonists may in many cases be preferred for subjects with obesity, and although we attempted to adjust for this, we did not have direct measurements of BMI.

Propensity-score matching is a method of mimicking some of the characteristics of a randomized controlled trial (34); i.e., the propensity score is a balancing score which guarantees the same distribution of observed baseline characteristics between two groups if subjects have the same propensity score. The caliper width was set according to previous studies on minimizing bias with propensity-score matching (35), and we obtained a fairly balanced matching, although the age distribution was not balanced out.

Furthermore, the matching process resulted in the discarding of a large number of subjects; the cohort reduced from 27,543 to 18,380 individuals. Hence, a sensitivity analysis was performed on the full cohort to examine whether any bias was introduced or efficiency lost in the matching process.

In addition, this study performed a variety of subgroup and sensitivity analyses, almost all of which point towards no difference in fracture risk between the two treatments. This robustness of the results supports the conclusion of neutral effects on fracture with SGLT2 inhibitor treatment compared to GLP-1 receptor agonist treatment in this population.

As this was an observational study, residual confounding cannot be ruled out. Particularly, we were unable to account for diet and exercise, both of which might be associated with the exposure (as obesity may influence the choice of glucose-lowering drug) and with the outcome. Lack of access to lab results and other clinical information meant that data on glycemic control, BMD, BMI, and other markers of significance to bone health were not available to be adjusted for. As such, we did not have information on vitamin D status or vitamin D supplementation prior to or during the study period, which poses a limitation to the study. However, although vitamin D status is causally connected to the outcome of the study, we do not expect a causal relationship between baseline vitamin D status and choice of SGLT2-i vs. GLP-1 RA treatment; therefore, any association between vitamin D status and the choice of exposure drug is expectedly governed by underlying common causes, which we expect to have been adjusted for *via* the other covariates. In addition, of the covariates we did include in the model, some were crude proxy-variables, e.g., obesity, smoking, and alcohol consumption. Similarly, data on falls and other risk factors for fracture were limited, as the utility of diagnosis codes to identify such factors is limited.

The relatively recent introduction of SGLT2 inhibitors in 2012 and the unavailability of outcome data after 2018 meant relatively short follow-up periods in the study. As fractures are in part a result of poor bone health, and changes in bone structure appear slowly, it is not certain that a differential effect on fracture risk would manifest during the study period. However, in the matched cohort, a full 9,153 individuals had at least one year of follow-up time, with 4,961 of those having more than two years.

Arguably the most important limitation of this study is the relatively small number of fractures (171 MOF in total in the main analysis), which is linked to the relatively short follow-up period. However, as all HRs found were below 1.00 (and the upper bounds of the confidence intervals close to 1.00), it is unlikely that a harmful effect of SGLT2 inhibitors has been overlooked, whereas a slight protective effect cannot be ruled out entirely.

## Conclusion

Overall, the results indicate no effect on fracture risk with SGTL2 inhibitor treatment when compared to GLP-1 receptor agonist treatment. The study is in line with previous research and supports the continued use of both drugs in the management of T2D in patients at risk of (osteoporotic) fracture.

## Data availability statement

The data analyzed in this study is subject to the following licenses/restrictions: Access to the utilized registries can be applied for at Statistics Denmark by any authorized research institution. Requests to access these datasets should be directed to forskningsservice@dst.dk

## Author contributions

All authors contributed to the article according to the ICJME requirements for co-authorship. All authors critically revised the paper for intellectual content and approved the submitted versions and the final version of the manuscript. ZKA, RV, and JS-L designed the study. ZKA, RV, JS-L and PV had access to all data used in the study. ZKA performed data management and statistical analyses with assistance from all co-authors. ZKA and RV interpreted the data and wrote the manuscript. JSL, PV and SG made ongoing critical revisions of study design and data interpretation.

## Funding

This work was supported by a Steno Collaborative Grant, Novo Nordisk Foundation, Denmark (Grant no. NNF18OC0052064).

## Conflict of interest

The authors declare that the research was conducted in the absence of any commercial or financial relationships that could be construed as a potential conflict of interest.

## Publisher’s note

All claims expressed in this article are solely those of the authors and do not necessarily represent those of their affiliated organizations, or those of the publisher, the editors and the reviewers. Any product that may be evaluated in this article, or claim that may be made by its manufacturer, is not guaranteed or endorsed by the publisher.
